# Movidea: A Software Package for Automatic Video Analysis of Movements in Infants at Risk for Neurodevelopmental Disorders

**DOI:** 10.3390/brainsci10040203

**Published:** 2020-03-31

**Authors:** Walter Baccinelli, Maria Bulgheroni, Valentina Simonetti, Francesca Fulceri, Angela Caruso, Letizia Gila, Maria Luisa Scattoni

**Affiliations:** 1 R&D, Department, Ab.Acus srl, via F. Caracciolo 77, 20155 Milano, Italy; mariabulgheroni@ab-acus.com (M.B.); valentinasimonetti@ab-acus.eu (V.S.); 2 Research Coordination and Support Service, Istituto Superiore di Sanità, Viale Regina Elena 299, 00161 Roma, Italy; francesca.fulceri@iss.it (F.F.); angela.caruso@iss.it (A.C.); letizia.gila@iss.it (L.G.); marialuisa.scattoni@iss.it (M.L.S.)

**Keywords:** motion analysis, video signal processing, neurodevelopmental disorders, infant screening

## Abstract

Early detecting the presence of neurodevelopmental disorders plays an important role in the effectiveness of the treatment. In this paper, we present a novel tool to extract motion features using single camera video recordings of infants. The Movidea software was developed to allow the operator to track the movement of end-effectors of infants in free moving conditions and extract movement features automatically. Movidea was used by different operators to analyze a set of video recordings and its performance was evaluated. The results showed that Movidea performance did not vary with the operator, and the tracking was also stable in home-video recordings. Even if the setup allowed for a two-dimensional analysis, most of the informative content of the movement was maintained. The reliability of the measures and features extracted, as well as the easiness of use, may boost the uptake of the proposed solution in clinical settings. Movidea overcomes the current limitation in the clinical practice in early detection of neurodevelopmental disorders by providing objective measures based on reliable data, and adds a new tool for the motor analysis of infants through unobtrusive technology.

## 1. Introduction

Early detection of neurodevelopmental disorders is of paramount importance. Indeed, providing timely interventions during infancy maximizes the outcomes of the long-term prognosis of affected children, capitalizing on the high neuroplasticity characterizing this period of life [1].

Motor skills shown during infancy have been found to be predictors of cognitive impairments arising in later developmental stages [2,3], thus indicating motor assessment as a valuable tool to early detect signs of neurodevelopmental disorders in infants. 

Currently, in clinical practice, several tests are used to evaluate the motor performances of children at different ages. Nonetheless, such approaches suffer from major shortcomings. Some tests require the children to perform specific actions or to interact with objects [4,5], thus limiting their application to infants. Other tests rely on the subjective observation and rating of parents [6]. However, it should be noted that tests adopted depend on the subjective evaluation, rating, and experience of the examiner.

Another technique allowing the early detection of neuromotor diseases of infants is the Prechtl method of general movements (GMs) assessment [7]. GMs consist of complex movements in which all parts of the body participate. Typical general movements are characterized by complexity and variation, whereas atypical general movements exhibit a limited repertoire of movement variants [8]. There is wide consensus that GMs are expression of the young developing brain, and their quality is an index of the integrity of the developing cortical network [8]. Their assessment according to Pretchl’s method has been proven to predict cerebral palsy with a sensitivity greater than 91% and a specificity greater than 81% [9]. Moreover, GMs quality has also been associated with cognitive impairment, attention-deficit-hyperactivity disorder, and minor neurological dysfunction [10,11].

This method involves the qualitative evaluation by an expert observer of the features characterizing spontaneous general movements, recorded while the infant is in an awake calm state, lying in the supine position [12]. Even if GMs assessment is one of the most reliable methodologies for neurodevelopmental disorders detection, the need for a trained expert observer and the subjective and qualitative nature of the GMs assessment reduce the widespread and applicability of this assessment in daily clinical practice [13,14].

Technology-based automatic analysis of motor performances may represent a solution for providing low-cost objective evaluations. With this goal, different approaches have been proposed to track, quantify, and analyze the motor behavior in infants. 

Wearable sensors such as accelerometers [15] and electromagnetic tracking systems [16] have been used to estimate the motion of the infants’ limbs. These systems result in being too cumbersome to be applied to infants and require accurate calibration and positioning procedures. 

Optical motion capture systems have also been proposed [17] to perform movement analysis of children’s limbs. In [17], an optoelectronic system (6 cameras, 18 markers) was used to describe the movement of the infants. A set of metrics was computed on the basis of the extracted kinematic data, and the findings showed these metrics as being able to identify infants with spasticity correctly. Even if this approach ensures an accurate motion tracking and measurement, it requires devoted high-cost equipment and a time-consuming preparation process, making it not applicable outside of dedicated labs.

In [18], the kinematics of hand movements in infants was studied using video analysis to identify markers of neurodevelopmental disorders. Although the results showed that kinematics in infants with neurodevelopmental disorders present characteristics identifiable through video movement analysis of upper limbs, the applicability of the method was limited by the setup used. Two video cameras were needed to monitor a single limb, and a visual marker (i.e., wristband) was applied to the infants’ wrist for analysis, affecting the conditions of the recorded infants.

Another approach is presented in [19], where a 3D camera was used to capture RGB and depth information from infants lying on their back, and an anatomical model was used to fit the data and reconstruct the movement. The study showed the applicability of this approach to GMs analysis, but its actual usage requires very high computational power, the storage of a large amount of data, and the manual intervention of a technical expert. These limitations limit the transferability of this approach to everyday clinical practice. A review of the currently available technology used to perform movement analysis in newborns for assessing GMs investigated the automatic analysis of video recordings [20]. The potential of this technique relies on the high availability of commercial video cameras and in the large amount of information recorded.

In the present study, we introduce a novel software (Movidea) that is based on semi-automatic video-based analysis of infants’ motor performance. Movidea involves the tracking of infants’ limbs using video recordings acquired by a single camera and the extraction of features for the description and evaluation of infants’ motion during free movement conditions.

## 2. Materials and Methods

### 2.1. Video Database of the NIDA Network

The Italian Network for early Detection of Autism spectrum disorder (ASD) (NIDA network) is the largest Italian cohort of infants at risk for AS. The NIDA network enrolls high risk infants (i.e., siblings of children with a diagnosis of ASD, preterm newborns, and small for gestational age newborns) and low risk infants (i.e., siblings of typically developing children) after delivery with the aim of recording and assessing infant crying and spontaneous movements at 10 days, and 6, 12, 18, and 24 weeks of age. In addition, a comprehensive clinical evaluation of the infants/toddlers was performed at 6, 12, 18, 24, and 36 months. The study was carried out according to the standards for good ethical practice and the guidelines of the Declaration of Helsinki. The study protocol was approved by the Ethics Committee of the Istituto Superiore di Sanità (Approval Number: Pre 469/2016). Written informed consent from a parent/guardian of each participant was obtained.

The video recording of the infant’s movements was generally performed at home while the child was lying on a bed, upon a green blanket provided by the NIDA network. The camera was placed 50 cm above the child, at chest height. The recording took place for at least 5 min with the aim of acquiring images of spontaneous movement of the full body of the child. To be analyzed with Movidea, each video recording was edited offline. A preliminary analysis of the videos showed that the high-quality video of all segments (i.e., without external interferences) did not exceed 3 min. Thus, we decided to save a 3 min video segment that represented the shorter high-quality frame for each recording. One author cut each video to ensure the same properties: 3 min length, infant in supine position, in a condition of well-being and spontaneous motor activity, without crying episodes. If videos showed more than 3 min of high quality frame, we decided to analyze the first high quality 3 min. Videoframes containing interferences by the operator and parents, as well as accidental movements of the camera, were excluded from the analysis. 

For this study, 300 videos from the NIDA database were analyzed. A total of 90 infants were video recorded (mean gestational age at birth = 39.05 ± 1.35 weeks, mean body weight at birth 3300.98 ± 383.78 g, mean body length at birth = 50.27 ± 1.76 cm). Infant risk status, sex, and age at recording are reported in Table 1.

Infant risk status, sex, and age at recording using a 3D camera are reported in Table 2.

### 2.2. Movidea Software

Movidea develops upon the arising need to identify early markers of neurodevelopmental disorders in infants, obtained through objective measures taken outside the clinical settings. In order to respond to this need, the software was designed to extract kinematic features of limbs from single-camera video recordings acquired in free movement conditions. The features were computed using two different approaches. On one hand, the trajectories covered by the infant’s limbs during the free movement were extracted using a semi-automatic limbs’ tacking procedure. On the other hand, movement quantification was performed through image processing techniques applied to the video frames. The software was developed using MATLAB ver. R2017a and its standard tools. The Movidea software was implemented for and is owned by the Italian research governmental institution, Istituto Superiore di Sanità, and by the Ministry of Health that funded the NIDA Network project. The software was implemented exclusively for research purposes. 

**Figure 1:**
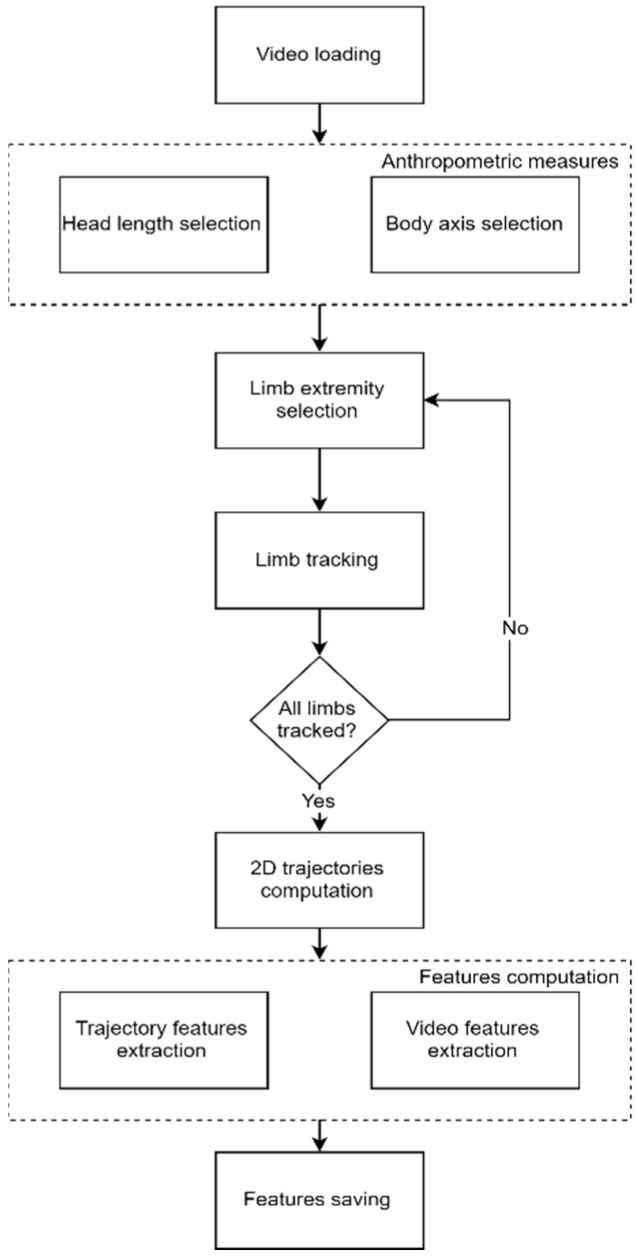
Movidea workflow.

The overall workflow of the software is reported in Figure 1.

The software was designed to allow the operators to go easily through the complete software workflow. A Graphical User Interface was developed to guide the software operator through each step. The operators were equipped with a user manual describing the software and all the interaction modalities, but no specific training was provided by technical experts. This aspect highlights the general usability of the software and easiness of operation deriving from the proposed approach.

### 2.3. Movement Tracking

The absolute distance could not be measured using one camera setup, and thus the 2D tracked trajectories needed to be measured in pixels. Indeed, the relation between the pixel and the actual distance measure depended on several factors such as camera resolution and camera–subject distance, making this relation not constant outside the single video framework. Thus, using the pixel as the measurement unit did not allow for the comparison of the data among different videos.

**Figure 2:**
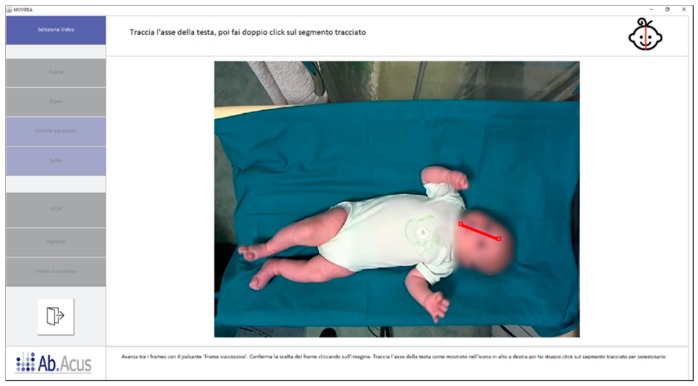
Head length line drawing. The red line connecting the forehead to the chin represents the head length measure taken by the operator.

To overcome this issue, the measure, in pixels, of the head length was used to normalize the data as anthropometric-related information suitable for allowing comparisons along time and subjects. The selection of the head length measure was the first step required by the software before proceeding with the tracking, and it was performed by manually setting the starting and the ending point of the line connecting the forehead and the chin of the infant in a video frame where both the points were clearly visible (Figure 2).

**Figure 3:**
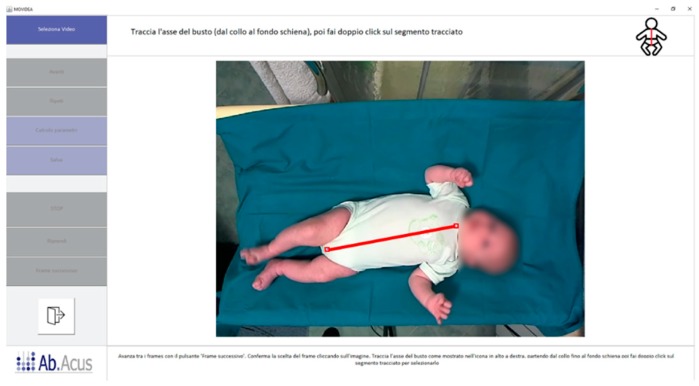
Body central line drawing. The red line connecting the clavicle-line mid-point to the inferior margin of the pelvis represents the body symmetry line taken by the operator.

Besides the head length, the operator was requested to select the central line of infant’s body (symmetry line) as the perpendicular line running down the surface of the body passing from the midpoint of the clavicle-line to the midpoint of inferior margin of the pelvis (Figure 3). This operation allowed the operator to compute the body orientation in the image frame and, therefore, to represent the trajectories with standard orientation and to perform a final visual check of the data quality.

Once the reference measures were taken, the limbs tracking can be performed. For each limb, the tracking required the operator first to identify the limb by selecting the central point of the end effector (i.e., hand, foot). The selected point was then tracked frame by frame using the Kanade–Lucas–Tomasi (KLT) algorithm [21]. To reduce the computational load and false positives, the algorithm was configured to search for the matching point in a squared area with a side size equal to 25% of the head length, centered in the coordinates of the point identified in the previous frame. In case the algorithm failed to locate the point in a frame, the operator could manually re-set the point to be tracked. If the tracked end effector was not visible in the frame (e.g., hidden by other body segments), the operator could skip the frame, avoiding producing invalid data.

**Figure 4:**
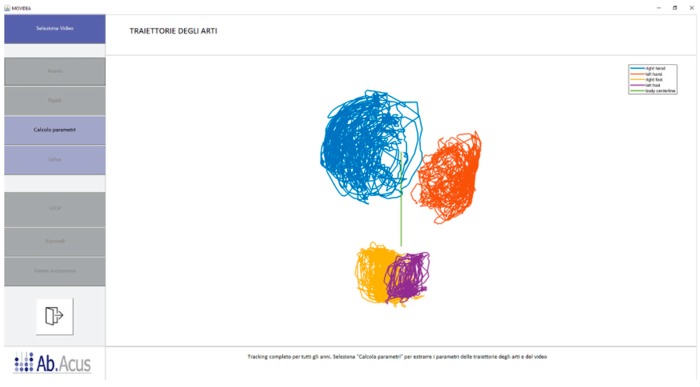
Trajectories represented here by lines of the four limbs tracked during an acquisition. Blue line = right hand; red line = left hand; yellow line = right foot; purple line = left foot; central green line = body symmetry line.

The result of the tracking process for each limb was a *N × 2* matrix containing the coordinates of the end effector’s reference point in the image for each of the *N* frames of the video (Figure 4).

The trajectories were then normalized by the head length, and a linear interpolation was applied to compensate the missing values corresponding to the skipped frames. Indeed, if a limb was not tracked for a long time period, the interpolation may produce an artificial trend in the data and may compromise the informative content. For this reason, the data were not interpolated in case the limb presented more than five consecutive missing values. As the sampling rate of the analyzed videos was 12.5 Hz, the maximum time interval for the interpolation of missing data was equal to 400 ms.

The preprocessed trajectories were used for the computation of a set of movement features meaningful for the identification of pathological motion patterns [17]:

*Velocity and Acceleration—*The velocity was computed for each limb as the Euclidian distance of the reference point’s location between two subsequent frames. The fast oscillations of the velocity profiles were then canceled through a third order low-pass Butterworth filter, with a cut-off frequency equal to the 95% of the Nyquist frequency. The acceleration of each limb was computed as the difference between two subsequent velocity samples. The mean velocity and mean acceleration of each limb was computed.

*Cross-correlation (CC)—*The zero-lag cross correlation between the velocity of each pair of limbs was computed as reported in [14], using the following equation:

(1) $$CC_{v1v2}=\frac{\sigma_{v1v2}}{\sqrt{\sigma_{v1}^{2}*\sigma_{v2}^{2}}}$$

where *CC_v_*_1*v*2_ is the cross-correlation between the velocity *v*1 and the velocity *v*2, σ*_v_*_1*v*2_ is the covariance of *v*1 and *v*2, $\sigma_{v1}^{2}$ is the variance of *v*1, and $\sigma_{v2}^{2}$ is the variance of *v*2. 

CC is a measure of the synchronicity of the movements of the limbs, and it is a suitable marker of neurodevelopmental disorders in infants [17].

*Area differing from moving average (A_ma_)—*For both the *x* and *y* components of the trajectory of each limb, the moving average was computed over the whole recording by using a window with a size of 30 samples according to the following equation:

(2) $${\bar{x}}_{i}=\frac{1}{k}\overset{i+\frac{k}{2}}{\underset{j=i-\frac{k}{2}}{\sum }}x_{j}$$

where ${\bar{x}}_{i}$ is the moving average computed at the *i*-th frame, *k* is the window’s size, and *x* is the point position in the *j*-th frame. 

The window size was chosen to average over 2 s, as reported in [17]. For each sample of the trajectory, the difference between the trajectory and the moving average was computed according to the following equation:

(3) $$A_{max}=\overset{l-\frac{k}{2}}{\underset{i=\frac{k}{2}}{\sum }}\left|x_{i}-{\bar{x}}_{i}\right|$$

where *A_max_* is the area differing from the moving average of the *x* component and *l* is the total number of frames of the recording. 

Moreover, the total *A_ma_* was calculated for the lower and the upper limbs as the sum of the area differing from the moving average of the two components of the two hands and the two feet, respectively. The *A_ma_* represents an index of the smoothness of the movements and it is a marker of neurodevelopmental disorders in infants [17].

*Periodicity (P)—*Periodicity is a parameter defined in [17] aimed at measuring the presence of repetitive movements in the motion of the limbs. To compute the periodicity, the recording was split into windows of 500 samples. In [17], the size of the window corresponded to one third of the total recording duration. To keep the computation coherent independently from the video length, the window’s size was chosen to guarantee the same time span of 40 s used in [17]. For both the components of the movement of each limb, the mean of the trajectory was computed over each window, and the intersections of the trajectory with the mean were detected. The mean distance $\bar{d}$ and the standard deviation $\sigma_{d}$ between consecutive intersections were computed. Finally, the periodicity *P* was computed by combining the parameters mentioned above, according to the following equation:

(4) $$P=\frac{1}{\bar{d}+\sigma_{d}}$$

### 2.4. Image Processing

The image processing approach leverages on the movement quantification from the changes occurring in the image from one frame to the next one. To this goal, the first step of the processing was the creation of motion images where only the pixels changed in one frame with respect to the previous one due to the infant’s movement were represented. In motion images, each pixel can assume only a value of 1 or 0, 1 (white) representing the occurrence of movement, and 0 (black) representing the absence of movement.

To obtain the motion images, the image of each frame was converted to black and white, and the difference with the black and white image of the previous frame was computed, resulting in a new image representing the changes occurring between the two frames. In order to account only for the changes related to the infant’s movement, a 2D median filter was applied to 5 × 5 pixel areas to remove salt and pepper noise. The pixels overcoming a predefined threshold were then set to 1, and all the other pixels were set to 0. The threshold was chosen as the optimal value for reducing the noise due to change in the light conditions and presence of blurry images, avoiding at the same time the suppression of actual movements of the limbs. For removing the residual noise present on the images, a convolutional filter with a 3 × 3 equally weighted kernel was finally applied. 

The motion images were used to compute several features related to the pathological conditions [22]:

*Quantity of motion (Q)*—is the number of pixels where the movement has occurred, divided by the total number of pixels in the image. The mean (*Q_mean_*), the standard deviation (*Q_sd_*), and the maximum value (*Q_max_*) are computed [22].

*Centroid of motion (C)*—is a parameter representing the central point of the infant’s movement in a given motion image. C is computed as the centroid of the cluster resulting from the application of a one-cluster *k*-means to the movement pixels of each motion image. The mean values C_xmea_n and C_ymean_ of C in *x* and *y* directions are computed over the recording together with the standard deviations C_xsd_ and C_ysd_ [14]. The mean and the standard deviation of the velocity (V_mean_, V_sd_) and the acceleration (A_mean_, A_sd_) of the centroid are also computed.

### 2.5. Software Validation

In order to verify the independence of measures extracted from the operator, a subset of 10 videos was analyzed through Movidea by two independent users, sharing the same instructions on how to operate the software. 

The trajectories obtained by the scoring were compared between the two operators by computing the zero-lag correlation coefficient. Indeed, this approach allowed for a trend comparison rather than a comparison of the absolute position of the tracked point, which did not affect the final measures.

In addition, the consistency of the features extracted by the two operators was tested. To this scope, the intraclass correlation coefficient (ICC) [23] was computed using a two-way random single measure absolute agreement model [24]. The ICC was computed only for the features extracted from the trajectories, as the image processing features were automatically extracted and were independent of the operator intervention.

The tracking failure rate was computed as the percentage of the number of times the operator had to manually re-set the tracking point, with respect to the total number of frames. This score was computed on a sample of 300 analyzed video segments.

Another important issue to be verified involving assessing the methodology that was implemented in Movidea was the dimensionality of the information. The single camera setup resulted in a reduction of the three-dimensional motion of the limbs to a bidimensional space implying a reduction of information. Given these considerations, it is useful to quantify the information loss. For this purpose, we recorded five infants’ videos using a 3D camera (RealSense D435, Intel, Santa Clara, CA, USA). Through the 3D camera, the RGB video and the depth information were recorded. The depth and RGB images were registered to obtain the 3D coordinates of the recorded points. The RGB videos were analyzed using Movidea, and the tracked trajectories were mapped in the new 3D space. The features previously described were computed on the 3D trajectories. The *z*-axis contribution was estimated on the features computed on the single axes (i.e., *A_ma_*, $n_{int}$, $\bar{d}$, and *P*) as the percentage of the feature computed on *z* with respect to the sum of the features computed on *x*, *y*, and *z*.

## 3. Results

Movidea was successfully used by non-technical operators to analyze over 300 video segments of infants, without major issues reported and without the intervention of a technical expert.

The mean correlation coefficients were computed between the trajectories obtained by the two operators for each video analyzed. The mean values of the correlation coefficients are reported in Table 3.

The results show that the tracked trajectories were highly correlated and, thus, the tracking procedure was stable across different operators.

The ICC coefficients reported in Table 4 were higher than 0.75 for all the features, indicating an excellent degree of agreement between the measures taken from the two operators [25].

The results of the analysis of the third-dimension impact reported in Table 5 show that the information loss due to the dimensionality reduction was 36.7% on average with a maximum of 53%, highlighting that the two-dimensions features accounted for most of the informative content, but that the analysis may have taken advantage of a three-dimensional data acquisition setup easily obtainable thanks to the wide availability of mainstream commercial RGB and depth cameras, their encumbrance, and costs.

Finally, in Table 6, the mean percentage of the tracking failures with respect to the total number of frames is reported for each end-effector.

## 4. Discussion

The goal of this paper was to evaluate if an automatic extraction of quantitative measures from video recordings could describe motor behaviors of infants. To this aim, we developed and tested a software implementing a semi-automatic analysis of movements in infants using single-camera video recordings. The software computes a set of features chosen according to their reported relevance in the literature and the occurrence of neurodevelopmental disorders. In particular, two different classes of features for the description of movement in infants were investigated: features extracted from the analysis of the trajectories of the limbs and features extracted from the analysis of movement images. The first class of features included the set of variables that in [17] were shown to be correlated with the occurrence of neurodevelopmental disorders. Such features relied on the extraction of infants’ kinematics from the sequence of images recorded in the video, as well as on the computation of parameters able to describe such kinematics. The second class of features implemented the metrics identified as predictors of the occurrence of neurodevelopmental disorders in [14] and in [22]. Differently from the first class of features, these parameters did not rely on kinematic information but take advantage of the changes in the sequence of images to infer information on the infant’s motion. 

Movidea software is a valuable tool for several reasons. First, the performed analysis showed that the implemented approach was user-independent, even if the operator had to interact with the software in the data extraction phase. The tracked trajectories and the features extracted did no vary when different users operated the analysis. This aspect is of paramount importance to assure the homogeneity of the measures when multiple operators elaborate a large amount of data. Second, the low percentage of failures in the tracking process showed that the tracking strategy implemented in Movidea well fitted recordings in real-life settings, allowing wide spreading of the method. Third, the choice to use a single camera approach highly enhances the usability of Movidea. Indeed, the use of unobtrusive and off-the-shelf technology may boost the uptake of technological solutions to investigate early motor development in populations at risk. The longitudinal assessment of motor functioning in populations at risk for neurodevelopmental disorder is worth exploring further because it may be useful in detecting social disorder or other developmental disorders [26,27,28]. By extracting meaningful information and objective, reliable data through a light setup and an easy to use tool, Movidea overcomes the current limitation, resulting in it being effectively applicable in multicentric and large population studies.

The results presented, nonetheless, showed that some information was lost due to the dimensionality reduction. Even if this loss did not compromise the validity of the approach, the use of 3D information may have added value to the analysis. To this purpose, an alternative solution for the data acquisition using a 3D camera combining RGB and depth information was proposed.

Overall, the results showed that Movidea is a reliable tool for the description of infants’ movements from 2D video recordings. This is a promising approach that raises attention to the automatic analysis of movement. Indeed, recent studies have proposed different tools for the analysis of video recordings of infants. For example, in [27], an explorative methodology for the pose estimation of joints of infants in video recordings was reported, whereas in [28], a platform was implemented for performing video recordings of infants and for extracting the velocity and the acceleration of the limbs. Nonetheless, these studies aimed at facilitating the visual inspection of the recordings for the identification of GMs. Movidea takes a step forward, producing a large set of features, both from kinematics analysis and motion images, with the aim of moving from a qualitative visual analysis to a quantitative analysis of infants’ movements.

## Figures and Tables

**Table 1:** Characteristics of infants video-recorded using a 2D camera.

Subjects		Age of Recording				
Risk	Sex	10 days	6 weeks	12 weeks	18 weeks	24 weeks
		*n*	*n*	*n*	*n*	*n*
Low risk	M	14	23	22	20	18
	F	8	15	16	9	11
High risk	M	13	14	16	16	13
	F	13	14	16	16	13

**Table 2:** Characteristics of infants video-recorded using a 3D camera.

Subject	Risk	Sex	Age of Recording
1	Low risk	F	12 weeks
1	Low risk	F	18 weeks
1	Low risk	F	24 weeks
2	Low risk	M	12 weeks
2	Low risk	M	24 weeks

**Table 3:** Trajectories’ correlation coefficients. For each axis of each limb, the mean ± SD of the correlation coefficients computed between the trajectories obtained by the two operators in each analyzed video is reported.

Limb	Axis	Correlation Coefficient
Right Hand	*x*	0.991 ± 0.004
	*y*	0.990 ± 0.005
Left Hand	*x*	0.992 ± 0.003
	*y*	0.980 ± 0.035
Fight Foot	*x*	0.989 ± 0.005
	*y*	0.966 ± 0.037
Left Foot	*x*	0.973 ± 0.028
	*y*	0.964 ± 0.034

**Table 4:** Intraclass correlation coefficients (ICCs) for the features extracted from the tracked trajectories. The ICC coefficients were computed using the features extracted from a set of five videos analyzed by two operators.

Feature	ICC
Mean velocity	0.98
Mean acceleration	0.99
Area from moving average	0.97
Cross-correlation coefficient	0.96
Intersections mean distance	0.87
Total number of intersections	0.94
Periodicity	0.97

**Table 5:** Contribution of *z*-axis to the total. For each feature, the mean ± SD contribution of the *z*-axis to the feature value is reported.

Feature	Name	*z* Contribution (%)
*A_marh_*	Area from moving average right hand	36.7 ± 3.4
*A_malh_*	Area from moving average left hand	41.6 ± 5.5
*A_marf_*	Area from moving average right foot	37.9 ± 4.4
*A_malf_*	Area from moving average left foot	35.7 ± 1.4
${\bar{d}}_{rh}$	Intersections mean distance right hand	16.8 ± 6.9
${\bar{d}}_{lh}$	Intersections mean distance left hand	11.3 ± 1.1
${\bar{d}}_{rf}$	Intersections mean distance right foot	16.5 ± 6.2
${\bar{d}}_{lh}$	Intersections mean distance left foot	18.0 ± 4.3
$Tin_{rh}$	Total number of intersections right hand	44.0 ± 10.0
$Tin_{lh}$	Total number of intersections left hand	53.9 ± 2.5
$Tin_{rf}$	Total number of intersections right foot	45.9 ± 10.8
$Tin_{lf}$	Total number of intersections left foot	43.4 ± 8.1
$P_{rh}$	Periodicity right hand	46.2 ± 10.1
$P_{lh}$	Periodicity left hand	52.4 ± 1.7
$P_{rf}$	Periodicity right foot	49.1 ± 11.8
$P_{lf}$	Periodicity left foot	47.2 ± 12.7

**Table 6:** Mean ± SD percentage of tracking failures. For each tracked limb, the percentage of frames in which the operator reset the tracking point is reported.

End-Effector	Failure (%)
Right hand	9.7 ± 6.7
Left hand	10.3 ± 6.7
Right foot	15.2 ± 9.3
Left foot	14.5 ± 9.2
